# Emotional freedom techniques for elderly patients with COVID-19: a case series on clinical recovery, frailty, and inflammatory biomarkers

**DOI:** 10.3389/fpsyg.2025.1627592

**Published:** 2025-09-11

**Authors:** Nina Kemala Sari, Erlina Burhan, Fathiyah Isbaniah, Dewi Yennita, Stepvia Stepvia

**Affiliations:** ^1^Department of Internal Medicine, Geriatric Division, Dr. Cipto Mangunkusumo National Referral Hospital, Faculty of Medicine, Universitas Indonesia, Jakarta, Indonesia; ^2^Department of Pulmonology and Respiratory Medicine, Persahabatan Hospital, Jakarta, Indonesia; ^3^Department of Clinical Pathology, Persahabatan Hospital, Jakarta, Indonesia

**Keywords:** emotional freedom techniques (EFT), COVID-19, elderly, IL-6, frailty, WHO ordinal scale

## Abstract

**Background/Objectives:**

Older adults are disproportionately affected by COVID-19 due to immunosenescence and comorbidities, resulting in higher rates of severe illness and mortality. Psychological distress such as anxiety and fatigue further compounds disease burden. Emotional Freedom Techniques (EFT), an integrative psychophysiological approach, has shown potential in enhancing psychological resilience and modulating inflammatory responses.

**Methods:**

We report a case series of five elderly patients with confirmed COVID-19 admitted to Persahabatan Hospital, Jakarta. Each received standard pharmacological care, including antiviral therapy, corticosteroids, and comorbidity management, alongside daily EFT sessions combining acupressure, affirmations, and exposure techniques. Clinical symptoms, frailty status (via WHAS criteria), WHO Ordinal Scale for Clinical Improvement (OSCI), and serum IL-6 levels were monitored over a 30-day follow-up period. Emotional well-being was qualitatively assessed through follow-up interviews and therapist observations of patient engagement and affective behavior.

**Results:**

All patients demonstrated substantial clinical improvement. OSCI scores decreased from baseline values of 3–4 to 1 by the final follow-up, representing a 66–75% reduction in clinical severity. Frailty status improved markedly across all cases. IL-6 levels showed an average reduction of approximately 85%, suggesting a clinically meaningful improvement in systemic inflammation. No residual symptoms or adverse events were reported. Patients also demonstrated enhanced emotional well-being and adherence to EFT through digital guidance.

**Conclusion:**

EFT may serve as a safe and supportive adjunct therapy in elderly COVID-19 patients, potentially accelerating clinical recovery and reducing inflammation and frailty. Further controlled trials are warranted to evaluate broader applicability in geriatric care.

## Background

In December 2019, reports emerged from Wuhan, China, describing a novel virus that caused symptoms ranging from mild respiratory illness to severe pneumonia and acute respiratory distress, later Identified as coronavirus disease 2019 (COVID-19) or SARS-CoV-2 (Cocuzzo et al., 2022; Shi et al., 2020). The pandemic has had a profound impact on older adults, who experience higher rates of morbidity and mortality due to immunosenescence, frailty, and underlying comorbidities (Jachymek et al., 2022; Crudeli et al., 2022; Tana et al., 2023b). Among biomarkers linked to disease severity in elderly COVID-19 patients, interleukin-6 (IL-6)—a pro-inflammatory cytokine—plays a key role and has also been associated with the progression of frailty (Promislow and Anderson, 2020; Wang et al., 2020).

Mortality from COVID-19 increases dramatically with age, from 0.1% in infants to nearly 15% in older adults, highlighting a more than 100-fold increase in risk across the human lifespan (Promislow and Anderson, 2020; Wang et al., 2020). Given this heightened vulnerability, adjunctive therapies that can reduce physical and psychological burden are urgently needed. In the post-vaccination phase of the pandemic, a growing number of patients, particularly older adults, report persistent symptoms that significantly impair daily functioning. This condition, known as long COVID, is defined by continuation or emergence of new symptoms following the acute phase of SARS-CoV-2 infection (Tana et al., 2022; Tana et al., 2023a).

The Emotional Freedom Techniques (EFT) is an integrative psychophysiological intervention that combines elements of exposure therapy with acupressure. It involves structured steps such as setup statements, tapping on specific meridian points, and optional affirmations aimed at reducing psychological and physiological distress. Rather than being a variant of cognitive behavioral therapy, EFT is a somatic approach focusing on emotional regulation through tactile stimulation (Bach et al., 2019; Sari et al., 2017).

A study in Indonesia with elderly patients indicated that Autosuggestion therapy, a component of EFT, correlated with enhanced quality of life and increased immunological function. This led to decreased levels of interleukin-6 (IL-6) (Tambunan et al., 2022). IL-6 is a pro-inflammatory cytokine often elevated in severe COVID-19 and frailty syndromes in older adults. Other studies have demonstrated that EFT can reduce anxiety and depressive symptoms in people diagnosed with COVID-19 (Tambunan et al., 2022). Mechanistically, Yount et al. (2019) found that EFT modulated gene expression related to cytokine production, while Bach et al. (2019) reported reductions in cortisol and resting heart rate following EFT sessions. A recent systematic review by Church et al. (2022) summarized the physiological effects of EFT, including reductions in inflammatory markers, stress hormones, and modulation of the autonomic nervous system.

This case series aims to explore the feasibility and potential effects of EFT as an adjunctive therapy in older adults with COVID-19, focusing on clinical severity, frailty, and systemic inflammation.

## Methods

This case series included elderly patients (aged ≥65 years) with confirmed COVID-19 who were admitted to the Department of Pulmonology at Persahabatan Hospital, Jakarta, Indonesia, between September 2022 and February 2023. Inclusion criteria were RT-qPCR-confirmed SARS-CoV-2 infection, mild to moderate disease severity according to the WHO Ordinal Scale for Clinical Improvement (OSCI), and willingness to participate in adjunctive EFT. Patients requiring intensive care or with cognitive impairments were excluded.

All patients received individualized standard care, including symptom-based pharmacologic treatment (e.g., analgesics, antipyretics, expectorants, antihistamines, decongestants, mucolytics, bronchodilators), antiviral agents (favipiravir or remdesivir, as indicated), multivitamin supplementation, corticosteroids (dexamethasone or methylprednisolone), and management of comorbidities such as hypertension, diabetes, and chronic kidney disease.

In addition to standard pharmacologic care, EFT was introduced as a daily self-help practice under the guidance of trained healthcare professionals. EFT followed a simplified Clinical EFT protocol adapted for elderly patients in hospital and home settings. Each session lasted 5–10 min and involved three steps: (1) setup statements identifying emotional or physical distress, (2) tapping on eight specific acupressure points on the face and upper body while verbalizing distress-related phrases, and (3) the inclusion of positive affirmations once distress had subsided. Formal use of Subjective Units of Distress (SUD) scores or individualized trauma-focused processing was omitted to improve adherence and accessibility (Bach et al., 2019; Sari et al., 2017). Patients received instruction through in-person guidance, video demonstrations, and printed modules. Daily digital reminders via the BotMD® application were used to promote adherence during hospitalization and follow-up. Family members were also trained to assist during recovery. No specialized equipment was required.

Primary outcomes included changes in clinical severity (OSCI), frailty status (using the Women’s Health and Aging Study/WHAS criteria), and inflammatory markers (IL-6 and C-reactive protein/CRP). OSCI is a standardized 0–10 scale that measures illness severity based on clinical status, ranging from asymptomatic (0) to death (10) (Rubio-Rivas et al., 2022). It encompasses the need for oxygen, hospitalization status, and symptom severity. WHAS frailty criteria assess physical domains such as weight loss, exhaustion, weakness, slow walking speed, and low activity. Measurements were taken at baseline (hospital admission), at 2 weeks, and at 1 month post-discharge. Emotional well-being was assessed through structured interviews but not using validated psychometric scales.

This study received approval from Ethics Committee of Persahabatan Hospital (Reg. No. 81/KEPK-RSUPP/09/2022). Informed consent was obtained from all patients or their legal representatives.

## Case series

### Case 1

A 68-year-old woman was admitted to the Department of Pulmonology at Persahabatan Hospital, Indonesia, presenting with high-grade fever, persistent cough, and headache. Three days prior to admission, she developed a fever (39 °C), which persisted despite self-administration of paracetamol. She subsequently visited her primary care provider and was referred for further evaluation. Upon arrival, her vital signs were: temperature 39.4 °C, blood pressure 160/78 mm Hg, heart rate 90 bpm, respiratory rate 20 breaths/min, and oxygen saturation 93% on room air. Her anthropometric measurements were 80 kg and 162 cm, yielding a BMI of 30.5. Physical examination revealed a dry cough and bilateral pulmonary rales; other systems were unremarkable.

She had a medical history of hypertension, diabetes mellitus, and thyroid dysfunction. Frailty was assessed using the WHAS criteria, yielding a score of 4. Laboratory investigations showed: hemoglobin 14.1 g/dL, hematocrit 42.5%, leukocytes 8,480/μL, platelets 224,000/μL, erythrocytes 4.63 million/μL, RDW-CV 13.9%, MCH 30.4 pg., MCHC 33.2 g/dL, MCV 91.7 μm^3^, basophils 0.4%, eosinophils 3.3%, neutrophils 74.1%, lymphocytes 10.6%, monocytes 11.6%, SGOT 98 U/L, SGPT 54 U/L, urea 15 mg/dL, creatinine 0.7 mg/dL, IL-6 3.2 pg./mL, and CRP 22.7 mg/dL. RT-qPCR testing confirmed COVID-19, with an OSCI score of 3.

In addition to standard medical treatment, the patient was introduced to EFT. A trained healthcare provider offered instruction supported by an educational video for self-guided practice. Family members were involved to reinforce adherence during hospitalization. Daily follow-up and reminders were facilitated via the BotMD® application. Clinical evaluations were performed at 2 weeks post-initiation, and subsequently on a monthly basis.

Starting from the second follow-up, the patient indicated that all symptoms had completely resolved, including fever, respiratory issues, fatigue, anosmia, and other COVID-related symptoms. At the third visit, IL-6 levels were 3.6 pg./mL, the OSCI score improved to 1, and frailty decreased to a score of 2. By the fifth visit, IL-6 decreased further to 2.8 pg./mL, CRP reduced to 2.0 mg/dL, and both the OSCI and frailty scores reached 1 and 0, respectively.

### Case 2

A 71-year-old woman presented to the Department of Pulmonology at Persahabatan Hospital, Indonesia, with a 5-day history of cough and dyspnea. The patient’s daughter had recently been diagnosed with COVID-19. Concerned about her symptoms and recent exposure, the patient sought medical care. On presentation, her vital signs were: temperature 39 °C, blood pressure 125/70 mm Hg, heart rate 84 bpm, respiratory rate 24 breaths/min, and oxygen saturation 92% on room air. She weighed 62 kg and was 158 cm tall, resulting in a BMI of 24.8. On examination, thick breath sounds, bilateral crackles, and wheezing were noted; other findings were within normal limits.

Her past medical history included chronic kidney disease. WHAS criteria yielded a frailty score of 3. Laboratory parameters revealed: hemoglobin 10.2 g/dL, hematocrit 30.6%, leukocytes 13,000/μL, platelets 286,000/μL, erythrocytes 3.35 million/μL, RDW-CV 14.1%, MCH 30.4 pg., MCHC 33.4 g/dL, MCV 91.1 μm^3^, basophils 0.3%, eosinophils 0.6%, neutrophils 78.6%, lymphocytes 13.7%, monocytes 6.9%, SGOT 27 U/L, SGPT 14 U/L, urea 40 mg/dL, creatinine 1.2 mg/dL, and IL-6 51.5 pg./mL. CRP was not measured. A subsequent RT-qPCR confirmed COVID-19, with a disease severity classified as OSCI score 4.

Alongside standard pharmacologic therapy, the patient was enrolled in the EFT program. A healthcare professional provided instruction and an educational video for independent practice. Family members monitored adherence, and daily prompts were delivered via BotMD®. Evaluations were conducted at the two-week mark and subsequently every month.

By the second follow-up, the patient was asymptomatic. At the third evaluation, IL-6 had declined significantly to 2.4 pg./mL, with an OSCI score of 1 and a frailty score of 2. At the fifth visit, IL-6 was 2.9 pg./mL, CRP was 1.5 mg/dL, the OSCI score remained 1, and the frailty score improved to 1.

### Case 3

A 79-year-old woman was admitted to the Department of Pulmonology at Persahabatan Hospital, Indonesia, presenting with dry cough, fatigue, and nausea. These symptoms had persisted for 7 days, accompanied by low-grade fever and mild dyspnea. Her caregiver reported close contact with a confirmed COVID-19 case who had died 2 weeks prior to the patient’s admission. On physical examination, her vital signs were as follows: temperature 38.5 °C, blood pressure 155/85 mmHg, heart rate 75 beats per minute, respiratory rate 18 breaths per minute, and oxygen saturation 94% on room air. Her body weight was 40 kg and height 154 cm, resulting in a BMI of 16.8, indicating undernutrition. Auscultation revealed bilateral wet crackles at the lung bases. Past medical history included hypertension and diabetes mellitus. Her frailty status, assessed using the WHAS criteria, yielded a score of 4.

Initial laboratory findings were as follows: hemoglobin 11.4 g/dL, hematocrit 29.9%, leukocytes 7,420/μL, platelets 156,000/μL, erythrocytes 3.44 million/μL, RDW-CV 11%, MCV 87.1 fL, MCHC 38 g/dL, neutrophils 86.3%, lymphocytes 7%, monocytes 6.6%, SGOT 16 U/L, SGPT 14 U/L, urea 11 mg/dL, creatinine 0.4 mg/dL, IL-6 37.1 pg./mL, and CRP 106.7 mg/dL. RT-qPCR confirmed SARS-CoV-2 infection. Disease severity was categorized as moderate with an OSCI score of 3.

EFT was introduced as an adjunct to standard pharmacological management. The patient was trained by a healthcare provider and supported with video instructions for daily self-practice. Family members were involved to encourage adherence. Daily reminders were sent via the BotMD® application. Follow-up evaluations were conducted at 2 and 4 weeks.

By the second visit, the patient reported full symptom resolution. At the third visit, IL-6 decreased to 6.8 pg./mL, OSCI score improved to 1, and frailty score reduced to 1. At the final follow-up, IL-6 was 6.6 pg./mL, CRP decreased to 1.9 mg/dL, and both OSCI and frailty scores remained stable at 1.

### Case 4

A 65-year-old man presented with a 4-day history of fever, myalgia, and productive cough. He had recently visited his grandson who was diagnosed with COVID-19. At admission, his temperature was 38.7 °C, blood pressure 138/82 mmHg, heart rate 88 beats per minute, respiratory rate 22 breaths per minute, and oxygen saturation 94% on room air. His BMI was 26.4 (72 kg, 165 cm). Lung auscultation revealed coarse breath sounds and crepitations in the lower lobes.

Medical history included asthma and dyslipidemia. Frailty assessment (WHAS) indicated a score of 3. Laboratory data showed: hemoglobin 13.6 g/dL, hematocrit 41.2%, leukocytes 9,800/μL, platelets 232,000/μL, RDW-CV 13.3%, MCV 89.2 fL, neutrophils 76.2%, lymphocytes 15%, monocytes 7.8%, SGOT 34 U/L, SGPT 28 U/L, urea 18 mg/dL, creatinine 0.8 mg/dL, IL-6 25.5 pg./mL, and CRP 19.6 mg/dL. RT-qPCR confirmed COVID-19, with a corresponding OSCI score of 3.

EFT was initiated alongside standard COVID-19 therapy. An instructional video was provided by a healthcare professional for independent practice. Family supervision supported adherence throughout.

During the second follow-up, the patient indicated complete resolution of systemic symptoms, including the absence of fever, respiratory issues, lethargy, anosmia, and other COVID-related manifestations. At the third visit, IL-6 had declined to 7.3 pg./mL, OSCI score to 1, and frailty score to 2. At the fifth visit, IL-6 was 3.2 pg./mL, CRP 1.4 mg/dL, and frailty score normalized to 0.

### Case 5

A 74-year-old man presented with persistent fever, mild chest discomfort, and anosmia that began 1 week prior. He reported recent close contact with a caregiver who tested positive for COVID-19. At admission, his vital signs were: temperature 38.9 °C, blood pressure 142/88 mmHg, heart rate 82 beats per minute, respiratory rate 20 breaths per minute, and oxygen saturation 91% on room air. He weighed 66 kg and measured 160 cm tall (BMI 25.8). Chest auscultation revealed decreased breath sounds at the right base.

His medical history included hypertension and hyperuricemia. The WHAS frailty assessment scored 4. Laboratory results included: hemoglobin 12.8 g/dL, hematocrit 38.5%, leukocytes 8,900/μL, platelets 215,000/μL, MCV 90.3 fL, neutrophils 72.9%, lymphocytes 17.1%, monocytes 9.3%, SGOT 22 U/L, SGPT 19 U/L, urea 16 mg/dL, creatinine 1.0 mg/dL, IL-6 29.7 pg./mL, and CRP 33.4 mg/dL. RT-qPCR confirmed SARS-CoV-2 infection, and OSCI score was assessed at 4.

In addition to standard treatment, the patient was enrolled in the EFT program. An instructional video and guidance were provided by a healthcare professional for independent practice.

By the third follow-up, the patient reported full symptom resolution. IL-6 levels dropped to 9.1 pg./mL, frailty score improved to 2, and OSCI decreased to 1. At the fifth follow-up, IL-6 was 4.0 pg./mL, CRP 2.2 mg/dL, and frailty score improved to 1.

All five elderly patients described in this case series demonstrated consistent clinical improvement following the application of EFT as an adjunct to standard COVID-19 treatment. Each patient initially presented with varying degrees of frailty, elevated IL-6 levels, and respiratory symptoms typical of COVID-19. After 1 month of daily EFT practice, all patients showed reductions in frailty scores, improved OSCI scores, and decreased levels of IL-6 and CRP. No residual (long COVID) symptoms were reported at the final follow-up. A comprehensive summary of baseline and final values for the OSCI, frailty status, and IL-6 levels across all five patients is presented in Table 1.

**Table 1:** Clinical, functional, and biomarker outcomes of EFT intervention in elderly COVID-19 patients.

Patient	OSCI		Frailty		IL-6	
	Baseline	Final	Baseline	Final	Baseline	Final
Case 1	3	1	4	0	3.2	2.8
Case 2	4	1	3	1	51.5	2.9
Case 3	3	1	4	1	37.1	6.6
Case 4	3	1	3	0	25.5	3.2
Case 5	4	1	4	1	29.7	4.0

OSCI, WHO Ordinal Scale for Clinical Improvement; IL-6, Interleukin-6 (expressed in pg/mL); Frailty was assessed using the Women’s Health and Aging Study (WHAS) criteria.

## Discussion

This case series highlights the potential benefits of EFT as an integrative intervention for elderly patients hospitalized with COVID-19. All five patients demonstrated improvements across clinical, functional, and biological parameters—specifically, reductions in OSCI scores, decreased frailty levels, and lowered inflammatory marker IL-6. These improvements may be explained by both physiological and psychosocial mechanisms, including reductions in psychological distress and systemic inflammation, enhanced coping mechanisms, and improved rest and adherence to care protocols.

Frailty is a key determinant of poor outcomes in COVID-19, often driven by immune dysregulation and persistent inflammation—particularly elevated IL-6 levels (Promislow and Anderson, 2020; Wang et al., 2020). All patients in this study were initially frail, but none remained frail at one-month follow-up based on WHAS criteria. This finding aligns with the etiopathogenesis of frailty described by Uchmanowicz et al. (2020), which includes biological (e.g., inflammatory), clinical (e.g., multimorbidity), and social contributors. Psychological interventions like EFT may buffer against these stressors by reducing neuroendocrine activation and improving emotional resilience.

EFT is a structured, low-cost mind–body technique that integrates cognitive processing with acupressure tapping. It has been shown to reduce cortisol levels and enhance vagal tone, contributing to improved autonomic regulation (Bach et al., 2019; Sari et al., 2017). These physiological changes support emotional stabilization, which may further enhance recovery in frail elderly individuals. Studies by Bach et al. (2019) and Church et al. (2012) demonstrated that EFT significantly reduces cortisol and enhances parasympathetic activity (Bach et al., 2019; Church et al., 2012), while a more recent study by Rochma et al. (2023) confirmed EFT’s efficacy in reducing anxiety. Given that anxiety can impair immune function and increase susceptibility to infections, these effects are particularly valuable in hospitalized geriatric patients.

In this case series, patients presented with varied symptoms, with cough and dyspnea being the most common. At day 30, all patients reported complete clinical recovery with OSCI scores of 1. Compared to a cohort study by Kumar et al. (2023), which showed that 90% of patients were discharged with OSCI scores of 0–2 and 4.3% had died by day 30, our series shows potentially faster recovery and zero mortality. Although this study lacks a control group, the findings suggest that the addition of EFT may have contributed to improved outcomes.

Reductions in IL-6 were consistently observed among all patients. IL-6 is both a biomarker of inflammation and a predictor of frailty, cognitive decline, and poor functional performance in older adults (Evans et al., 2021; Kappelmann et al., 2021; Rehman et al., 2017). Elevated IL-6 has also been associated with depressive symptoms and long COVID manifestations. A study by Puzianowska-Kuźnicka et al. (2016) confirmed that IL-6 levels increase with age and are inversely correlated with physical performance. Our findings are in line with previous EFT studies showing reductions in inflammatory markers like IL-6 and cortisol alongside decreased anxiety (Bach et al., 2019; Church et al., 2012; Hamidah et al., 2025), supporting a psychoneuroimmunological model in which emotional regulation promotes immune recovery.

While affirmations are sometimes included in later rounds of EFT, they are not core components of the Clinical EFT protocol. Their role in reinforcing adaptive beliefs may be especially beneficial in elderly patients facing prolonged illness or hospitalization. EFT facilitated emotional reconstruction, strengthened psychological well-being, and reduced stress, contributing to overall recovery and reduction in frailty and OSCI scores (Javidi and Fatahian Tehran, 2021).

Despite the positive outcomes, limitations must be acknowledged. This case series cannot establish causality, and improvements may be due to natural recovery or concurrent therapies. Emotional outcomes were not measured with standardized instruments, and biological markers were limited to IL-6. Nonetheless, the consistency of patient improvement and alignment with prior mechanistic evidence suggest that EFT may serve as a useful adjunctive therapy.

In conclusion, EFT appears to be a feasible, acceptable, and potentially effective integrative approach to support recovery in frail elderly patients with COVID-19. Its incorporation into geriatric care pathways may help bridge the gap between psychological and immunological recovery, offering a scalable strategy in both acute and post-acute settings.

## Data Availability

The raw data supporting the conclusions of this article will be made available by the authors, without undue reservation.
